# Rotational-invariant speckle-scanning ultrasonography through thick bones

**DOI:** 10.1038/s41598-021-93488-y

**Published:** 2021-07-09

**Authors:** Siyi Liang, Lidai Wang

**Affiliations:** Department of Biomedical Engineering, City University of Hong Kong, 83 Tat Chee Ave, Kowloon, Hong Kong SAR China

**Keywords:** Biomedical engineering, Applied physics

## Abstract

Ultrasonography is a major medical imaging technique that has been broadly applied in many disease diagnoses. However, due to strong aberration and scattering in the human skull, high-resolution transcranial ultrasonic imaging remains a grand challenge. Here, we explore the rotational-invariant property of ultrasonic speckle and develop high-resolution speckle-scanning ultrasonography to image sub-millimeter-sized features through thick bones. We experimentally validate the rotational invariance of ultrasonic speckle. Based on this property, we scan a random ultrasonic speckle pattern across an object sandwiched between two thick bones so that the object features can be encoded to the ultrasonic waves. After receiving the transmitted ultrasonic waves, we reconstruct the image of the object using an iterative phase retrieval algorithm. We successfully demonstrate imaging of hole and tube features sized as fine as several hundreds of microns between two 0.5 ~ 1-cm-thick bones. With 2.5-MHz excitation and the third-harmonic detection, we measure the spatial resolution as 352 µm. Rotational-invariant speckle-scanning ultrasonography offers a new approach to image through thick bones and paves an avenue towards high-resolution ultrasonic imaging of the human brain.

## Introduction

Ultrasonography is a major medical imaging technique offering high-resolution anatomical and functional information. Taking advantage of low cost, label-free, high speed, and deep penetration in soft tissue, ultrasonic imaging has been widely used in many anatomical sites. However, due to strong aberration/multiple scattering in the thick skull, ultrasonography has not been extensively applied to the human brain.

Several techniques, such as time-reversal^1,2^, speckle brightness^3,4^, and pitch-catch methods^5^, have been developed based on phase correction towards solving the aberration problem in the skull. The time-reversal technique can mitigate weak aberration but become ineffective for strong aberration. The speckle brightness method uses an iterative phase compensation algorithm to improve beamforming quality. The pitch-catch method corrects skull-induced phase abbreviations in pulse-echo images. Both techniques are restricted to a thin layer of bone near the transducer. O’Reilly et al.^6^ employed a passive imaging technique and low ultrasonic frequency to reduce scattering. The use of low frequency leads to a poor spatial resolution of several millimeters^7^. Via choosing a thinner and less aberration region, Lindsey et al.^8^ have developed a helmet-like imaging system to reduce aberration at the cost of a compromised resolution. Ultrasonic imaging through the human skull at high resolution still remains an open challenge. Singular value decomposition (SVD) beamformer is also proposed to correct aberration effects^9^.

Some imaging methods utilizing multiple scattering have also been proposed^10^. These methods exploit coherence along the array response matrix. These authors then term this antidiagonal coherence as ‘memory effect’^11^.

Here, we explore a rotational-invariant property of ultrasonic speckle. Contrary to ‘memory effect’ in^11^, this property is not operated in frequency domain. Our finding may be related to van Cittert-Zernike (VCZ) theorem^12,13^. Based on this property, we develop a new imaging technique, named rotational-invariant speckle-scanning ultrasonography, to acquire high-resolution ultrasonic images through thick bones.

## Principle of methods

The rotational-invariant property is described as follows. When an ultrasound wave passes through a strong acoustic scattering medium, we can obtain a random but stable speckle pattern. Tilting the incident beam by a small angle causes rotation or translation of the speckle pattern behind the scattering medium, and the speckle pattern remains almost the same within a certain angular range. Using the rotational-invariant property, we can scan the random speckle pattern behind strong scatterers.

When scanning the ultrasonic speckle pattern across an object, we can detect the total backscattered or transmitted signal $I$ as a function of the incident angle $\theta$ as

$$ I\left( \theta  \right) = \smallint O\left( r \right)S\left( {L\theta  - r} \right)dr = \left( {O*S} \right)\left( \theta  \right) $$

where* r* is the position in the region of interest, $L$ is the distance between the scatterer and the object, $O\left( r \right)$ denotes the object function, and $S\left( {L\theta  - r} \right)$ denotes the speckle pattern at the incident angle $\theta$ and the distance $L$, and $*$ denotes the spatial convolution. Taking autocorrelation of the intensity profile $I\left( \theta  \right)$, we obtain

$$ \left| {I\,\star\,I} \right| = \left| {\left( {O*S} \right)\,\star\,\left( {O*S} \right)} \right| $$

where $\,\star\,$ denotes autocorrelation operation, $\left|  \cdot  \right|$ denotes ensemble average of different speckle realizations. According to the property of autocorrelation of convolution^14^, i.e., $\left( {a*b} \right)\,\star\,\left( {a*b} \right) = \left( {a\,\star\,a} \right)*\left( {b\,\star\,b} \right)$, we can obtain

$$ \left| {I\,\star\,I} \right| = \left( {O\,\star\,O} \right)*\left| {S\,\star\,S} \right| $$

Ensemble averaged autocorrelation of random speckle patterns $\left| {S\,\star\,S} \right|$ is a Dirac delta function. Thus, we can simplify the above equation as

$$ \left| {I\,\star\,I} \right| = O\,\star\,O $$

In such a way, we can obtain the autocorrelation function of the original object, from which we can further recover the image of the object using a Gerchberg–Saxton-type iterative phase retrieval algorithm^15^.

To reconstruct the object function, we calculate Fourier transform of the autocorrelation of the measured signal $I$ to obtain the magnitude of the object’s power spectrum, i.e. $F\left[ {O\,\star\,O} \right] = ~F^{2} \left[ O \right]$, where $F$ denotes the Fourier transform. The object function can be reconstructed from its magnitude and phase spectra. The phase spectrum is lost but can be estimated using the phase retrieval algorithm. First, a random pattern $Ph_{0} \left[ O \right]$ is assigned as the initial phase. Then an object function value can be calculated from the magnitude and the phase spectra. Because the object function value is always real and positive, we can use this constraint to tune the next phase pattern $Ph_{i} \left[ O \right]$ so that the retrieved object function fulfills the real and positive constraint. Repeating this process, the retrieved results will gradually converge to the true object function.

## Materials and methods

### Experiment set-up

Marrowbone bought from market is cleaned and chopped into the proper size and ground with sandpaper sheets with 1000 grits (9083NA-20, 3 M) to a desired thickness, animals/humans were not directly involved in the study. To validate the memory effect for scattered ultrasound: A lab-made 2.5-MHz focused transducer is connected to a Pulser-Receiver (5072PR, OLYMPUS) and put in a water tank filled with deionized water, a rotation stage (KSP-606 M, SIGMAKOKI) is fixed at the bottom of the water tank. Previously made bone is fixed at the axis of rotation. The Pulser-Receiver is also connected to a digital oscilloscope (TBS1104, Tektronix). Echoes are observed on the oscilloscope when adjusting the rotation stage. Relative change of the amplitude should be small otherwise the position between the transducer and bone should be adjusted. After the tunning is done, the transducer is connected to an arbitrary waveform generator (DG4062, RIGOL). A phased array transducer (P4-1, ATL) is also fixed at a distance to the bone and make sure they rotate to the same point/axis. The transducer array is 10.5-cm away from the bone. We rotate the emitting transducer around its focal point. The rotational step size is 0.23 degrees. At every angle, 5 frames of ultrasound radio frequency (RF) data is collected.

For the 2.5-MHz pulse emission, we set a 300-mV amplitude, 8-cycle number, and a 5-ms duration as the stimulation signal and the sampling rate is 10 MHz.

The first imaged objects are printed with a Stratasys Fortus 400mc System using photosensitive resin (model 9400). During experiments, it is fixed with the rotation system together with another bone close to the phased array. A high power amplifier (LZY-22 + , Mini-Circuits) is connected to the arbitrary waveform generator and then to the focused transducer to ensure the signal-to-noise ratio. The object is kept 10.5-cm away from bone 1 and the scanning step size is 0.116 degrees. During tube imaging experiments, homemade microbubbles are pumped into a capillary tube using a syringe pump, then the tube is sealed. This capillary tube is kept 4-cm away from Bone_in. The phased array position is also adjusted to several millimeters away from Bone_out. The sampling frequency is 24 MHz to acquire the third harmonic signal according to Nyquist Theorem. A digital band-pass filter centered at 7.5 MHz (40-kHz passband) is applied to the RF data. The scanning step size is 0.0775 degrees.

### Signal processing

In the tube imaging experiment, the RF signal collected includes background signal and harmonic signal, a Matlab-implemented Kaiser-window FIR filter with 40-kHz passband is used to extract harmonic signal for further processing.

### Phase-retrieval algorithm

The phase-retrieval algorithm was implemented. The object constraints used were it being real and nonnegative. The algorithms were implemented in Matlab. A single run of the algorithm (composed of 3000 iterations) takes less than 1 s. The reconstructed results of Fig. 2 are the reconstruction results after median-filtering with a 3-pixel window.

## Results

**Figure 1:**
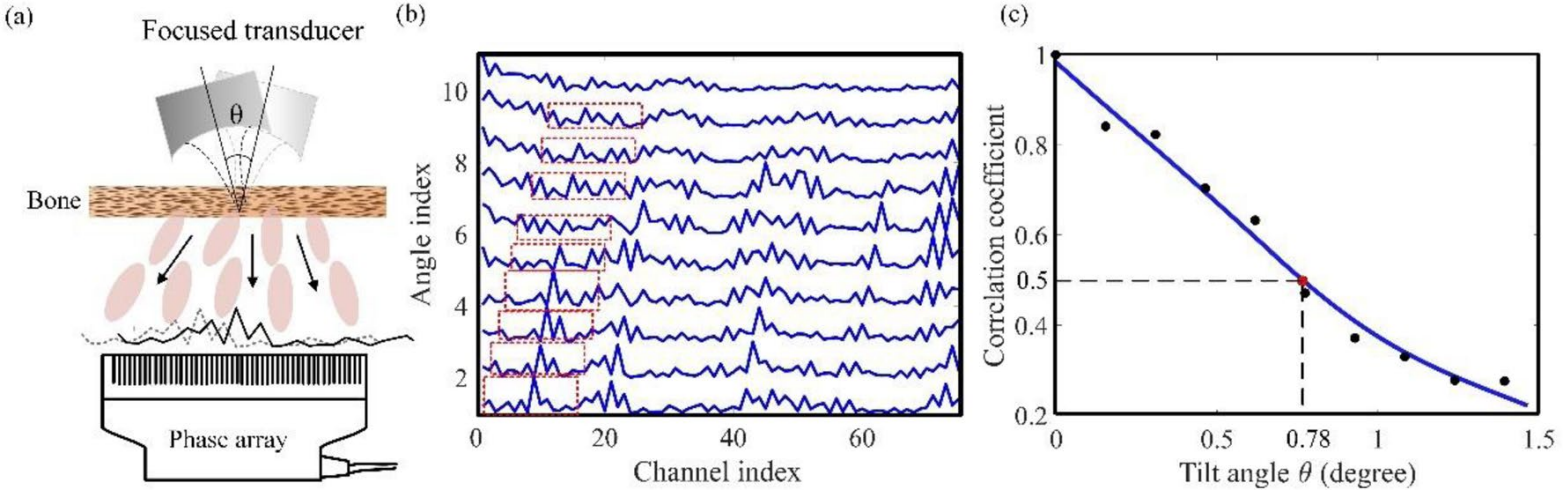
Experimental validation of rotational invariant ultrasonic speckle through bones. (**a**) Experiment setup. A single-element ultrasonic transducer focuses on a 1-cm-thick swine trabecular bone. A 96-channel ultrasound array transducer (P4-1, ATL) measures 1D speckles at 11-cm behind the bone. (**b**) Speckle patterns at different tilt angles. The x-axis is the channel index of the array transducer. The angular interval is 0.15 degrees per rotational step. Features in the dashed boxes validate the rotational-invariant property. (**c**) Cross-correlation coefficients between the first and other speckle patterns in (**b**). Data are smoothed by the spline.

We experimentally validate the rotational invariant property of ultrasonic speckles. Figure 1a shows the experiment setup. A customized narrow-band focused transducer emits 2.5-MHz continuous ultrasonic wave. The transducer has a diameter of 4.1 cm and a focal length of 2.4 cm. A piece of swine trabecular bone (1-cm thick) is placed in the transducer’s focal plane. Behind the bone, 1D ultrasonic speckle patterns are detected by a 96-channel ultrasonic array transducer (P4-1, ATL). The transducer array is 11-cm away from the bone. We rotate the emitting transducer around its focal point. The rotational step size is 0.15 degrees. The pitch of the ultrasonic transducer array is 0.3 mm. Considering the 11-cm distance between the bone and the ultrasonic array, the 0.15-degree angular step size corresponds to a translational step size of 0.29 mm in the measurement plane.

At each incident angular position, RF data in all channels are recorded for 5 s at a sampling frequency of 6 MHz. The mean square value of the RF data in each channel is calculated as the ultrasonic intensity. The intensity profile of all 96 channels (1D speckle) is normalized to their maximum value. Ten sets of 1D speckle patterns obtained at ten rotational angles in a full range of 1.4 degrees are shown in Fig. 1b. These 1D profiles exhibit obvious invariant patterns during the rotational scanning of the incident ultrasonic beam. Curves in the dashed box in Fig. 1b is a representative case. The 1D speckle patterns remain almost unchanged within certain angular range and then gradually decorrelates. Cross-correlation coefficients between the first and the other speckle patterns are shown in Fig. 1c, validating the rotational invariance. We define the angular range when the cross-correlation coefficient is no less than 50% to be the rotational-invariant range. Because the rotation in Fig. 1c is unidirectional, the full rotational-invariant range is twice of the range in Fig. 1c. In this experiment, the rotational invariant range is ~ 1.6 ± 0.1 degrees (mean and standard error, averaged 30 times). Eventually, the cross-correlation coefficient drops to ~ 0.2, but not 0. We think the reason is that the transmitted ultrasonic waves include both ballistic and scattered components, and the ballistic components remain correlated even in a large angular range.

In imaging experiments, we find that even when the cross-correlation coefficient is less than 0.5 (but greater 0.2), the speckle scanning still allows us to reconstruct the object image. To enlarge the field of view, the minimum allowed cross-correlation coefficient in the following imaging experiments can be smaller than 0.5 but larger than 0.2. Moreover, the ballistic acoustic wave is a problem in the imaging experiments.

Based on the rotational-invariant property, we scan the ultrasonic speckle pattern across samples behind thick bones. The rotational-invariant range is first measured before each imaging experiment. Two small holes on an 8-mm-thick plate are placed 15.5-cm-away from the bone. The plate is printed with photosensitive resin (model 9400). The holes allow ultrasonic waves to pass, and a layer of 2-mm-thick sealed air in other areas of the plate blocks ultrasonic transmission.

**Figure 2:**
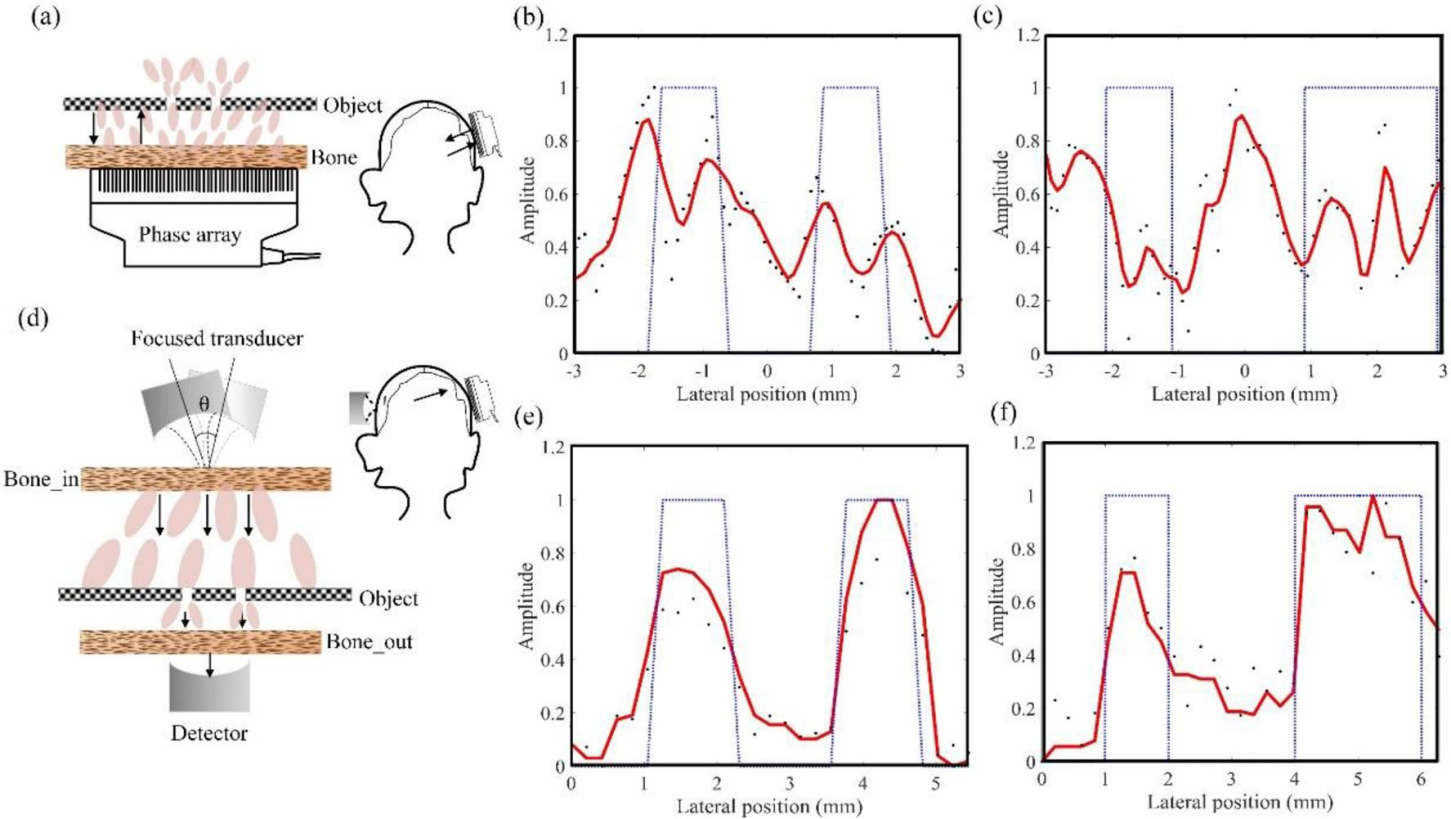
Imaging hole-features behind 1-cm-thick bones with conventional ultrasonography and rotational-invariant speckle-scanning ultrasonography. (**a**) Experimental setup for conventional ultrasonography. (**b**) and (**c**) 1D profiles of hole features acquired with conventional ultrasonography. True profiles are plotted as dashed lines for comparison. The object in (**b**) has two holes with 1-mm size and a 3-mm center-to-center distance. The object in (**c**) has two holes with 1-mm and 2-mm sizes, and a 3.5-mm center-to-center distance. (**d**) Experimental setup of rotational-invariant speckle-scanning ultrasonography. In experiment, an array is used as a detector. (**e**) and (**f**) Reconstructed 1D images of hole features and the ground truth. The objects in (**e**) and (**f**) are the same as the ones in (**b**) and (**c**).

For comparison, we first use conventional ultrasonography to image the hole features behind the 1-cm-thick bone, as shown in Fig. 2a. The ultrasonic array transducer (P4-1, ATL) has a 2.5-MHz central frequency and 73% bandwidth. Arrows indicate the emitting and receiving highly-scattered ultrasonic waves through the bone. We acquired B-mode images of two samples with different hole sizes (see supplementary information). To compare with the true hole features, 1D profiles of the B-mode images at the object positions are plotted in Fig. 2b and c. True 1D object profiles are plotted as dashed blue lines overlaid on the measured ultrasonic imaging profiles. In Fig. 2b, the object has two 1-mm-sized holes with a 3-mm center-to-center distance. In Fig. 2c, the object has two holes. The left one is 1 mm in size; the right one is 2 mm in size. The center-to-center distance is 3.5 mm. Figure 2b and c show strong variations due to aberrated/scattered ultrasound in the thick bone, and the hole features are invisible. As expected, conventional ultrasonography cannot image the fine features through the 1-cm-thick bone.

Figure 2d shows the experimental setup for rotational-invariant speckle-scanning ultrasonography. A single-element focused ultrasonic transducer emits 2.5-MHz continuous wave. The 1-cm-thick swine bone (labeled as Bone_in in Fig. 2d) is placed on the ultrasonic focus to scatter the ultrasonic wave. The object is placed 15.5-cm away from the bone (Bone_in). Another 0.5-cm-thick swine bone (labeled as Bone_out) is placed 2-mm away from the object. A flat receiving transducer is positioned 2-mm from the Bone_out. The aberrated or scattered ultrasonic waves transmit through the holes and then are detected by the receiving transducer. We use a 96-channel array transducer (P4-1, ATL) as the single-element flat transducer by adding all-channel data together without delay. The objects used here are the same as those in Fig. 2b and c. To image the objects with ultrasonic speckles, we rotate the emitting ultrasonic transducer around the acoustic focal spot and measure the transmitted ultrasonic intensities. The scanning step size is 0.0775 degrees. Ultrasonic intensities at 20 ~ 30 positions are acquired in each scanning. For ensemble averaging, after each scanning, we rotate the emitting transducer around the focal spot along the direction normal to the scanning direction to generate different speckle patterns. Then, we repeat the speckle scanning imaging using the new speckle pattern. We ensemble average the results from three random speckle realizations. We use the iterative phase retrieval algorithm to reconstruct the images from the averaged intensity data. Results are smoothed by moving average over three pixels and shown in Fig. 2e and f. The reconstructed images clearly show the hole features. Figure 2e shows two holes: the left one is 1.27 mm in full-width-half-maximum (FWHM), and the right one is 1.13 mm in FWHM. The peak-to-peak distance between them is 2.91 mm. In Fig. 2f, FWHM of the left hole is 1.07 mm, 2.20 mm for the right one. The peak-to-peak distance of the two holes is 3.77 mm. Compared with the ground truth, rotational-invariant speckle-scanning ultrasonography can successfully image the hole features. The imaged center-to-center distances (2.91 mm and 3.77 mm) are close to the true values (3 mm and 3.5 mm). In the images, the 2-mm hole is larger than the 1-mm holes. The imaged hole sizes are consistently larger than their true values by 0.07–0.27 mm.

We quantify the spatial resolution using thin line features. Thin glass tubes filled with microbubble solutions are placed between two bones. Microbubbles solutions in the tubes generate harmonic signals upon ultrasonic excitation^16,17^. In speckle-scanning ultrasonography, we detect the third harmonic signal to reconstruct the microbubble images so that the fundamental-frequency signal from the tube walls and other features can be suppressed.

**Figure 3:**
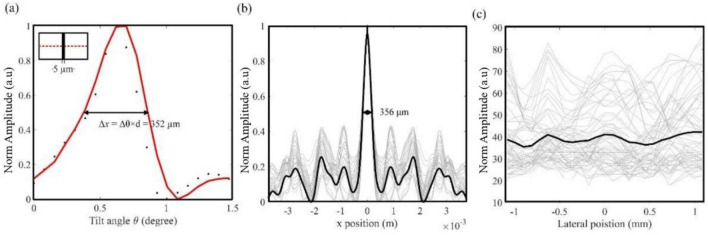
Speckle-scanning ultrasonography of capillary tubes between two 0.5 ~ 1-cm-thick bones. (**a**) Reconstructed image of a 5-$\mu m$-diameter tube filled with microbubbles. Full width at half maximum is 352 µm. (**b**) Gray: speckle autocorrelations of 20 sets of autocorrelation functions. Black: averaged autocorrelation function of speckle patterns in the object plane. (**c**). Conventional ultrasonography of the 5-$\mathrm{\mu }\mathrm{m}$-diameter tube. Gray: 1D profiles within field-of-interest (FOI). Black: an averaged 1D image. See Supplementary information.

The experimental setup is the same as the one shown in Fig. 2d. Lab-made lipid-shelled microbubbles have diameters of 1 ~ 5 $\mathrm{\mu }\mathrm{m}$. The resonate frequency is in the range of ~ 2–5 MHz. Microbubbles are mixed with water with a concentration of ~${10}^{9}$ bubbles per ml. The sampling frequency is 24 MHz to acquire the third harmonic signal. A digital band-pass filter centered at 7.5 MHz (40-kHz passband) is applied to the RF data. The scanning step size is 0.0775 degrees. The ultrasonic intensity profiles are averaged over six speckle implementations and then are used to reconstruct the object. To measure the lateral resolution, we image a capillary tube with 5 $\mathrm{\mu }\mathrm{m}$ inner diameter. The tube is placed at the other side of Bone_out. The distance is between them is 200 µm correspond to the tube wall. The distance between the object and Bone_out should guarantee that all signals received by the transducer are scattered twice by Bone_in and Bone_out. Currently, we validate the proposed method in the case when the distance between the object and Bone_out is as close as 200 µm. The reconstructed 1D image is shown in Fig. 3a. FWHM of the 1D profiles is 352 µm, which represents the lateral resolution.

The resolution of speckle-scanning ultrasonography is related to the speckle grain size in the object plane. The speckle grain size is measured from the FWHM of the averaged autocorrelation functions of the speckle patterns. In Fig. 3b, the averaged FWHM of the autocorrelation function is 356 ± 2 µm (mean and standard error, the sample size is 20) and is very close to the measured resolution. With the parameters used in our experiments, we can estimate the speckle-grain size using an equation in^18,19^. The calculated speckle-grain size is 339 µm, which is close to the measured resolution and speckle size. For comparison, conventional ultrasonography images the 5-µm tube through the 1-cm-thick bone. The conventional ultrasonic imaging result in the object plane is plotted in Fig. 3c. As expected, conventional ultrasonography cannot image the micro-tube feature through the thick bone even with the same number of averages.

The field-of-view (FOV) is determined by the angular range of rotational invariance and the distance between the object and the scatter (Bone_in). In experiments, we show a 6-mm imaging range with 25 effective pixels. To increase the imaging range and pixel number, further increasing the rotational-invariant range and the scanning step number is necessary. Optimizing the ultrasonic frequency or selecting a proper site on the skull may be helpful to further extend the rotational invariant range. Taking advantage of speckle scanning, the resolution of this technique can reach 352 µm, which overcomes the aberration/scattering issue in thick bones.

At present, we manually scan the ultrasonic transducer, and the time to acquire an image is around one hour. Automatic scanning or electronic beam steering in the future may dramatically shorten the imaging time. In addition, the imaging speed depends on the number of ensemble averages and the scanning steps. A fewer averaging number and a reasonable larger step size may shorten the imaging time.

As proof of concept, we only demonstrate 1D ultrasonic imaging through thick bones. As the speckle is fully developed, the spatial characteristic is uniform in the horizontal plane(correspond to the scan direction). So the extension from 1D speckle correlation scenario to 2D scenario is straightforward. This 1D method enables further development of 2D imaging technique. For instance, if we use the 1D imaging result as one projection, then we can acquire multiple projections at different angles and reconstruct a 2D image.

## Conclusion

We explore the rotational-invariant property of ultrasonic speckle through strong acoustic scatterers. The rotational-invariant angular range can be ~ 1.6 degrees when 2.5 MHz ultrasound penetrates through 1-cm-thick bones. Based on this property, we develop rotational-invariant speckle-scanning ultrasonography to image objects sandwiched between two 0.5 ~ 1-cm-thick bones. Speckle-scanning ultrasonography can image hole and line features that are invisible in conventional ultrasonography. With harmonic signals emitted from microbubbles, we quantify the lateral resolution as high as $352\,\mu\,m$. We expect this new technique enables high-resolution ultrasonography of the human brain.

## Supplementary Information

Supplementary Information.
