# Supplementary material for: Rotational-invariant speckle-scanning ultrasonography through thick bones
Source: Sci Rep. 2021 Jul 9;11:14178. doi: 10.1038/s41598-021-93488-y (PMC8270910; doi:10.1038/s41598-021-93488-y)
Supplement: Supplementary file 1 — Supplementary Information. [file 41598_2021_93488_MOESM1_ESM.docx]

Supplementary information for

Rotational-Invariant Speckle-Scanning Ultrasonography Through Thick Bones

Siyi Liang, and Lidai Wang^*^

Department of Biomedical Engineering, City University of Hong Kong, 83 Tat Chee Ave, Kowloon, Hong Kong SAR, China

^*^Corresponding to [lidawang@cityu.edu.hk](mailto:lidawang@cityu.edu.hk)

1. **Numerical simulation results**


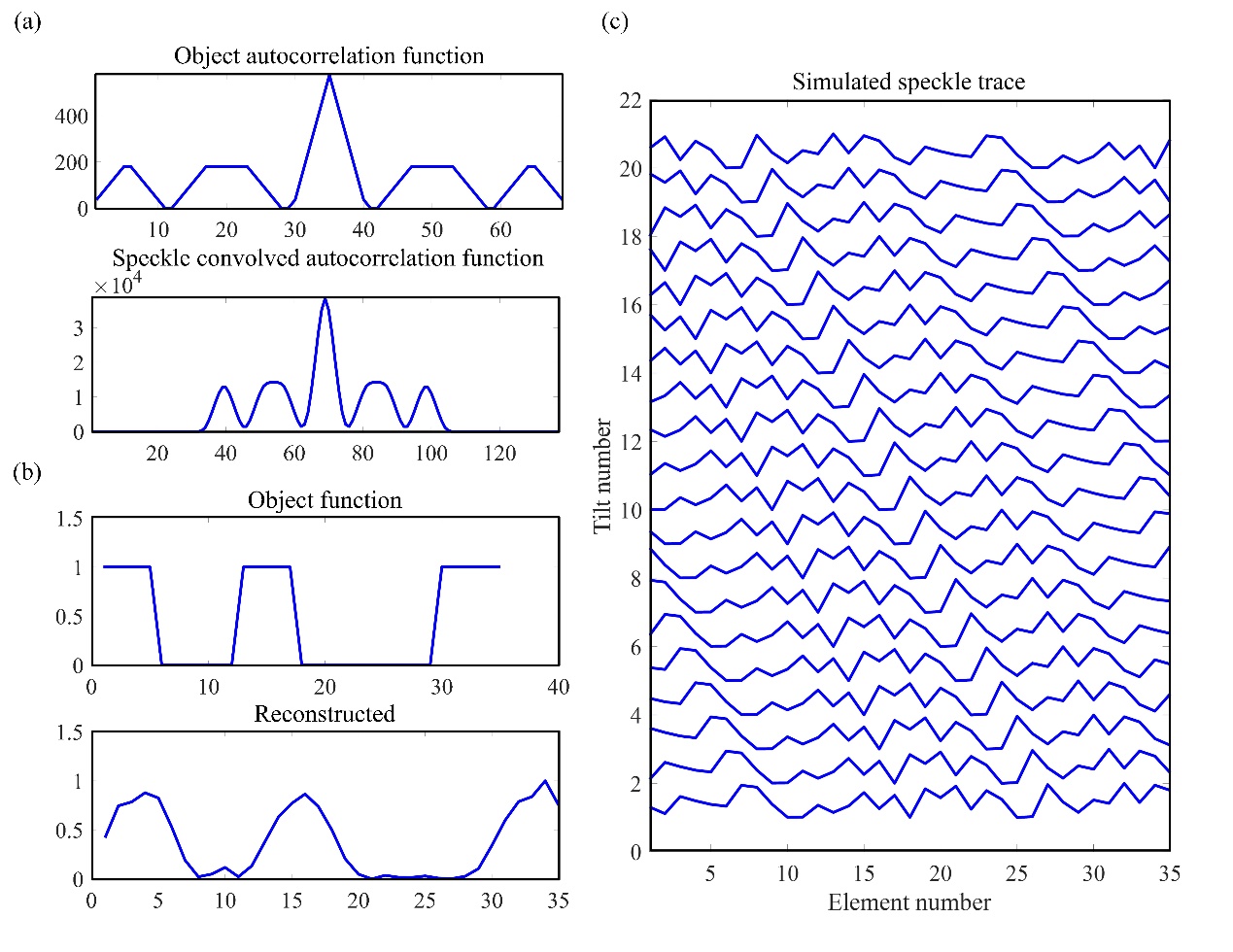


**Supplementary Figure 1: (a) Upper: autocorrelation of an object function. Lower: autocorrelation of the convolution between the object function and a random speckle. (b) Upper: true object function. Lower: reconstructed results by iterative phase retrieval algorithm. (c) Translating 1D random speckle patterns.**

To reconstruct an object function from its autocorrelation function, an iterative phase retrieval algorithm is adopted. In this algorithm, we use a constraint to iteratively retrieve the object phase. The constraint requires the reconstructed image pixels to be real and non-negative. Supplementary Figure 1.b plots the original object function. Figure 1.a gives the autocorrelation function of the object. To simulate the speckle-scanning process, we simulate a 1D translating speckle pattern as shown in Figure 1.c.

Convolution is performed between the speckle pattern and the object function. Then we calculate the autocorrelation of the convolution results and plot it in Figure 1a. The phase retrieval result is shown in Fig 1.b. Compared to the original object function, the reconstructed image can faithfully show the features of the object.

1. **Rotational-invariant speckle-scanning ultrasonography of hole features**
   1. **Imaging of two 1-mm-sized holes**

First, we scan the object with the rotation-invariant speckle patterns and collect the total energy transmitted. A representative 1D intensity profile is calculated and plotted in Supplementary Fig 2.


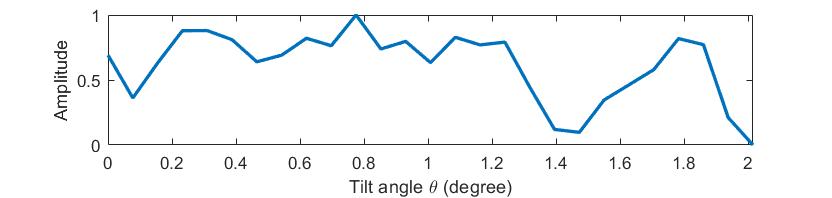


**Supplementary Figure 2: An intensity profile shows transmitted ultrasound energy at different incident angles.**

Eight intensity profiles are collected for the ensemble average. The autocorrelations of all intensity profiles are calculated and averaged. The ensemble average of different speckle patterns can average the speckle autocorrelation function, yielding an approximated Delta function. Supplementary Fig 3 shows the ensemble-averaged autocorrelation function, which is then used to retrieve the phase [1] and recover the image. The reconstructed image is shown in Fig. 2e.

**
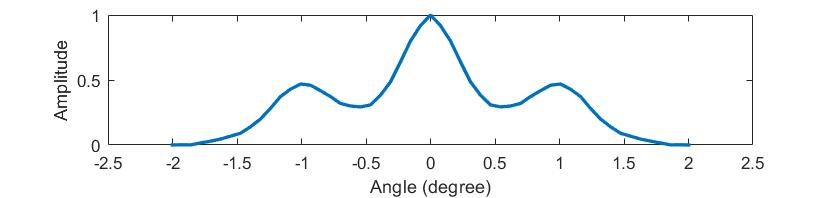
**

**Supplementary Figure 3: averaged autocorrelation from three different speckle realizations.**

- 1. **Imaging of 1-mm and 2-mm holes.**

The procedure to image an object with a 1-mm hole and a 2-mm hole is described as follows. The intensity profiles are acquired at different incident angles. One representative profile is shown in Supplementary Fig 4. Eight different speckle patterns scan across the object for the ensemble average. The ensemble-averaged autocorrelation function is calculated and plotted in Supplementary Fig 5. The autocorrelation result is used to reconstruct the 1D image in Fig. 2f.


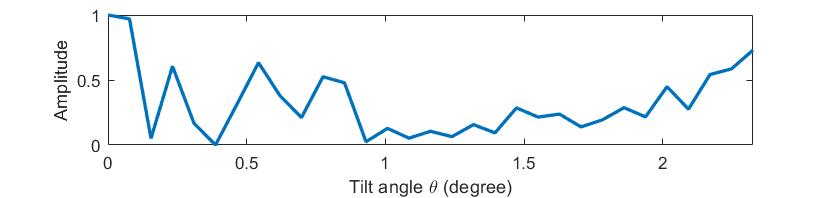


**Supplementary Figure 4: An intensity profile acquired at different incident angular positions.**


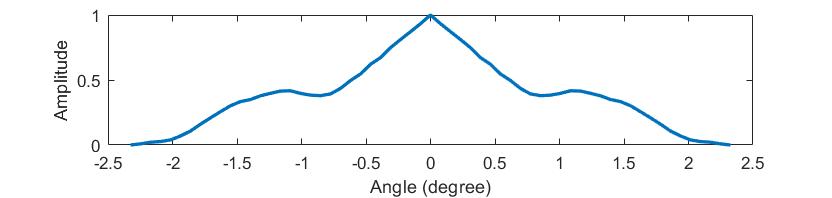


**Supplementary Figure 5: Averaged autocorrelation of three different speckle realizations.**

1. **Conventional ultrasonography of hole features**

Conventional ultrasonography pulse-echo B-mode images are shown here. An ultrasound array transducer (P4-1, ATL) and an ultrasound DAQ (Verasonics) are used to acquire the B-mode images.

- 1. **Imaging of two 1-mm holes**

A B-mode ultrasonic image containing the expected object location is shown in Supplementary Figure 6. Strong speckles which are originated from the bone appears in the image, but hole features cannot be absorbed. 1D profile along the expected hole position is plotted in Fig. 2b in the main text.


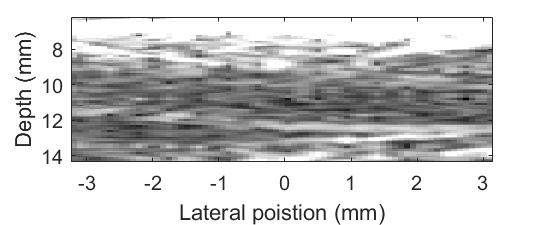


**Supplementary Figure 6: A B-mode image of two 1-mm holes. The hole features should be located around the image center.**

- 1. **Imaging of** **two holes of 1mm and 2mm respectively.**

A B-mode image of two holes (1-mm and 2-mm in size, 3.5-mm in the central distance) is shown in Supplementary Fig 7. A 1D profile of along the expected hole position is plotted in Fig. 2c in the main text.

**
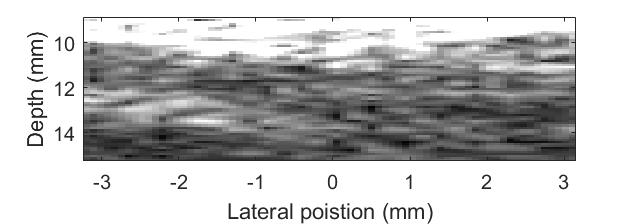
**

**Supplementary Figure 7: B-mode image of two holes of 1mm and 2mm in size. This image includes the expected hole features.**

1. **Conventional ultrasonography of a capillary tube**

A conventional B-mode ultrasonic image of a 5-µm-diameter tube is shown in Supplementary Figure 8. The expected cross-section feature of the tube is invisible in the B-mode image. A 1D profile at the expected tube position is plotted in Fig. 3c in the main text.


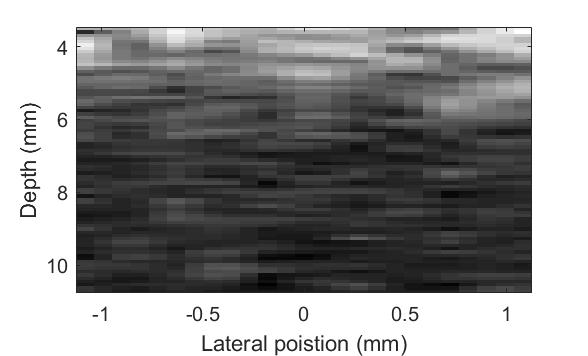


**Supplementary Figure 8: B-mode image of a 5-**$\boldsymbol{\mu m}$ **capillary tube filled with microbubbles**

1. **Acoustic speckle size calculation**

As discussed in [2], the speckle patterns only carry information about transducer and their focusing pattern. Both the intensity and magnitude of the speckle grain size are given.

In the transverse direction, the phasor magnitude

$$S_{cx\_m}= 0.87\lambda Z_{0}/D^{'}$$

The phasor intensity

$$S_{cx\_i}= 0.9\lambda Z_{0}/D^{'}$$

In the above equations, λ denotes the wavelength of the transducer, $Z_{0}$ denotes the distance from the transducer to the focal point. $D^{'}=D/1.08$ where $D$ denotes the diameter of the transducer.

Later in [3], the phasor magnitude equation is used as an average speckle spot size calculation. Here we also use this way of calculation. In our experiment, the transducer diameter is around 4cm, two-way time-of-flight from the focus is around 32 $\mu s$. The speed of sound in water is assumed 1500 m/s. Which gives us around 339 $\mu m$ average speckle spot size.

**Reference**

[1] J. R. Fienup, Optics letters **3**, 27 (1978).

[2] R. F. Wagner, IEEE Trans. Sonics & Ultrason. **30**, 156 (1983).

[3] S. Smith and R. Wagner, Ultrasonic Imaging **6**, 174 (1984).
